# Atrial septal defect and patent ductus arteriosus closure in an 8‐month‐old patient with Silver‐Russell syndrome

**DOI:** 10.1002/ccr3.4455

**Published:** 2021-07-19

**Authors:** Ryoma Oda, Keisuke Nakanishi, Shiori Kawasaki, Atsushi Amano

**Affiliations:** ^1^ Department of Cardiovascular Surgery School of Medicine Juntendo University Tokyo Japan

**Keywords:** atrial septal defect, cardiac surgery, patent ductus arteriosus, pulmonary vascular resistance, Silver‐Russell syndrome

## Abstract

We present a case of an 8‐month‐old boy with Silver‐Russell syndrome who had high pulmonary vascular resistance, atrial septal defect, and patent ductus arteriosus. He underwent cardiac surgery using cardiopulmonary bypass without any complications.

## Graphical abstract

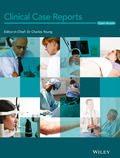

## 1 INTRODUCTION

Silver‐Russell syndrome is a rare genetic disease associated with severe prenatal and postnatal growth retardation. Little is known regarding cardiac surgery for Silver‐Russell syndrome. We present the case of an 8‐month‐old boy who underwent cardiac surgery; the postoperative course was good without pulmonary hypertensive crisis or other complications.

Silver‐Russell syndrome (SRS) is a rare but well‐recognized condition associated with pre‐ and postnatal growth retardation, dysmorphic facial features, and body asymmetry. Almost all patients with SRS are born small for their gestational age. The estimated incidence of this condition is approximately 1/30,000–1/100,000 live births.^1^ In 2016, the first international consensus statement regarding SRS was announced.^1^ There are many reports on growth disorders, growth hormone deficiency, mental retardation, hypoglycemia, and digestive disorders in patients with SRS, but few reports on congenital heart disease and surgical treatment in these patients. We report a rare case of an 8‐month‐old boy with SRS who underwent successful surgical repair with cardiopulmonary bypass.

## 2 CASE REPORT

The patient was an 8‐month‐old boy [weight, 5.85 kg (–2.5 SD); height, 65.7 cm (–3.0 SD)] who was born at 38 weeks gestational age (birth weight: 2116 g) with intrauterine growth retardation. He was diagnosed with an atrial septal defect (ASD) and a patent ductus arteriosus at birth. He had characteristic physical features, such as a triangular face, fifth finger clinodactyly, body asymmetry, and genital abnormalities. He was a low‐birth‐weight infant with these physical characteristics; therefore, we suspected a chromosomal abnormality. Genetic testing revealed 46, XY, del(7)(q32q34). The defective region contains the MEST gene, one of the imprinting centers on chromosome 7.^1^ Methylation analysis revealed that the methylation pattern was a maternal pattern; the deletion was in the paternal‐derived chromosome. Paternal‐derived deletion of this region has been reported to exhibit an SRS phenotype.^1^ Hence, we diagnosed SRS based on clinical features and genetic testing.

**FIGURE 1:**
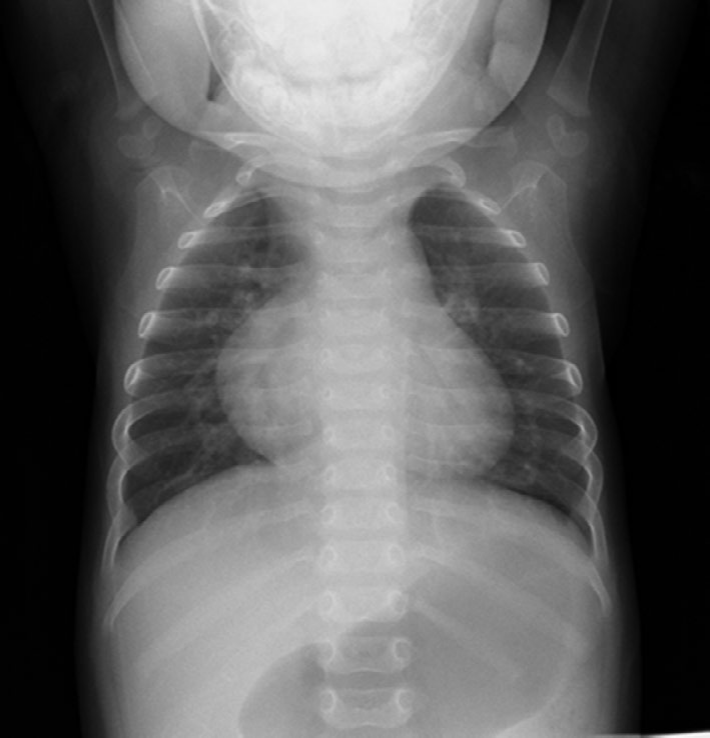
Preoperative chest radiograph demonstrating cardiac enlargement with enlargement of the right second arch and the left second‐to‐third arch

**FIGURE 2:**
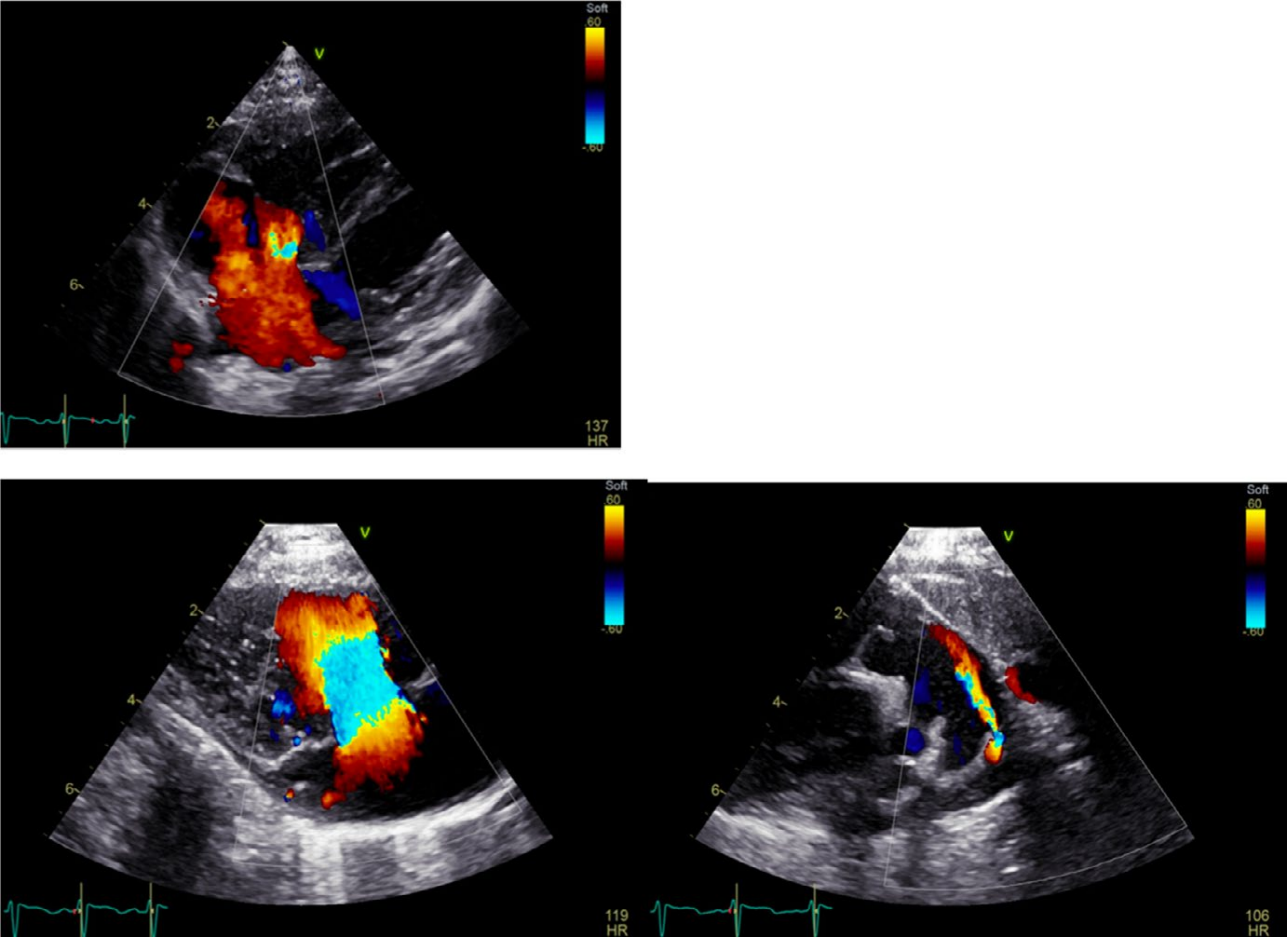
Preoperative echocardiography showing a prominent atrial septal defect jet and a patent ductus arteriosus

He had persistent pulmonary hypertension but no signs of heart failure, and brain natriuretic peptide (BNP) was unchanged at approximately 50 pg/ml; therefore, he was followed up on an outpatient basis (Figures 1, 2). He was admitted to the hospital at 6 months of age because of a lower respiratory tract infection, developed respiratory failure, and cyanosis. Echocardiography showed right ventricular enlargement; the estimated right ventricular pressure increased to 70 mm Hg, and the ASD shunt flow changed from left to right to right to left. BNP levels were high at 574 pg/ml. Diuretics and oxygen administration improved his symptoms, after which pulmonary artery catheterization was performed to evaluate shunt blood flow and pulmonary hypertension accurately. It revealed pulmonary hypertension, left‐to‐right atrial shunting, pulmonary blood flow/systemic blood flow ratio (Q_p_/Q_s_) = 2.67, the sum of left and right pulmonary artery cross‐sectional area/body surface area; PA index = 458, pulmonary vascular resistance value; R_p_ = 2.9–3.4 Wood Unit・m^2^, and no other cardiac anomalies. Simultaneous pressure measurement indicated that the aortic pressure was 100/75 mm Hg, and that the main pulmonary artery pressure was 60/20 mm Hg.

To prevent worsening of pulmonary hypertension, we decided to perform surgery early. The catheter intervention was not possible because he had low body weight and had porous ASD. At 8 months, he underwent surgery to close the ASD and patent ductus arteriosus to improve pulmonary hypertension.

**FIGURE 3:**
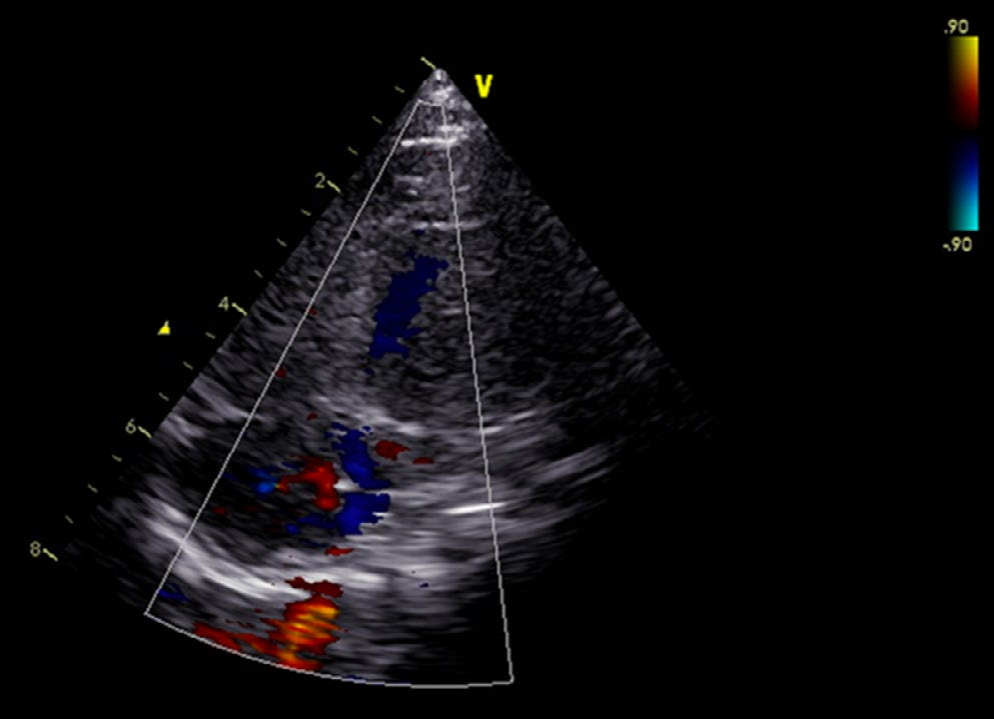
Postoperative echocardiography (six months later) demonstrating no atrial septal defect and no pericardial effusion

His body surface area was 0.37 m^2^. Surgical repair was performed through a median sternotomy. Before the cardiopulmonary bypass was established, PDA was identified and clipped. Cardiopulmonary bypass was established after heparinization just after pericardiotomy, and the right atrium was opened under mild hypothermic chemical cardiac arrest. A piece of pericardium was harvested. (20 × 15 mm) The patient had a type II ASD (ostium secundum type) that was cribriform and porous. After excising the inter‐atrial septum, the ASD was closed by a running suture using 5–0 polydioxanone (PDS [United State of America, Johnson and Johnson]) sutures and a pericardial patch. Intraoperative simultaneous pressure measurement was used to confirm that the right ventricular pressure was 26/6 mm Hg, and the aortic pressure was 97/69 mm Hg. After rewarming, the patient was easily weaned from cardiopulmonary bypass without arrhythmias. The total bypass time was 43 min, the cross‐clamp time was 15 min, and the operation time was 164 min. The postoperative hemodynamic state was stable without pulmonary hypertensive crisis. The patient's postoperative course was uneventful, and he was discharged on postoperative day 8. The postoperative echocardiogram showed no residual ASD and no pericardial effusion. Six months after the surgery, an echocardiogram showed no problems as well, and he is doing well as an outpatient (Figure 3).

## 3 DISCUSSION

It is crucial to decide if and when surgery should be performed in such young patients with chromosomal abnormalities and pulmonary hypertension. Our patient had preoperative pulmonary hypertension and a history of a transient right‐to‐left atrial shunt. Pulmonary hypertension may have been more advanced than we thought; therefore, we carefully evaluated the option of performing surgery. Only a few case reports have described the treatment of congenital heart disease associated with SRS.^2^, ^3^ To our knowledge, there are only two case reports of open‐heart surgery for SRS. One involves a school‐age patient that underwent surgery for ASD,^3^ and the other involves a neonate that underwent surgery for three atria.^2^ Our case involves ASD in an infant with preoperative transient right‐to‐left shunting and pulmonary hypertension. Some genetic diseases, such as trisomy 21, are associated with pulmonary hypertension's rapid progression. However, there are no such reports in patients with SRS.^4^ Preoperatively, our patient presented with temporary Eisenmenger‐like hemodynamics, which was accurately reevaluated by catheterization after treatment for heart failure. Based on its findings, we confirmed that the patient did not develop Eisenmenger physiology and that the pulmonary vascular resistance was not too high. Therefore, we performed early surgery to prevent pulmonary hypertension worsening using the standard procedure, and the patient showed a good postoperative course without pulmonary hypertension aggravation.

Another crucial point was performing the surgery safely with cardiopulmonary bypass. Vahlas et al.^3^ reported the first known case of successful ASD closure using cardiopulmonary bypass in a patient with SRS. They reported that the operation was performed via median sternotomy, complete mildly hypothermic cardiopulmonary bypass, and cardioplegic cardiac arrest.

We performed the operation with cardiopulmonary bypass in the same way as for any other infant in our hospital; we did not need any special procedures. The flow during cardiopulmonary bypass was based on the body surface area, calculated using the Mosteller formula, age, and weight nomograms. We calculated the patient's body surface area based only on his height and weight and did not consider age. The necessary flow rate was determined as follows: 2.6–2.8 × body surface area. The primary diagnosis did not influence the management strategy, which did not cause any complications related to cardiopulmonary bypass.

This case report shows that accurate hemodynamic assessment with careful consideration of the indications for surgery leads to good outcomes even in patients with SRS who have temporary Eisenmenger‐like hemodynamics before surgery. Many surgeons may be hesitant to operate because of the transient, preoperative right‐to‐left shunt and the possibility that the pulmonary vascular resistance may be more advanced than expected. Although SRS is also a genetic disease, like trisomy 21, there have been no reports of it being associated with a rapid progression of pulmonary hypertension. Based on our experience, we believe that the surgical indications and the intraoperative and postoperative management adopted for patients with SRS may be applicable for children without any genetic disease as well. We hope that this case report will be helpful to surgeons who may have to treat similar cases in the future.

## CONFLICT OF INTEREST

None.

## AUTHOR CONTRIBUTIONS

Ryoma Oda, MD: involved in article drafting. Keisuke Nakanishi, MD, PhD: involved in critical revision of the article. Shiori Kawasaki, MD, PhD and Atsushi Amano, MD, PhD: involved in approval of article.

## ETHICAL STATEMENT

We obtained informed consent verbally and documented it in the medical record. We also obtained approval from the ethics committee.

## Data Availability

The data that support the findings of this study are available on request from the corresponding author. The data are not publicly available due to privacy or ethical restrictions.
